# Dapivirine Bioadhesive Vaginal Tablets Based on Natural Polymers for the Prevention of Sexual Transmission of HIV

**DOI:** 10.3390/polym11030483

**Published:** 2019-03-13

**Authors:** Raúl Cazorla-Luna, Araceli Martín-Illana, Fernando Notario-Pérez, Luis-Miguel Bedoya, Paulina Bermejo, Roberto Ruiz-Caro, María-Dolores Veiga

**Affiliations:** 1 Departamento de Farmacia Galénica y Tecnología Alimentaria, Facultad de Farmacia, Universidad Complutense de Madrid, 28040 Madrid, Spain; racazorl@ucm.es (R.C.-L.); aracelimartin@ucm.es (A.M.-I.); fnotar01@ucm.es (F.N.-P.); rruizcar@ucm.es (R.R.-C.); 2 Departamento Farmacología, Farmacognosia y Botánica, Facultad de Farmacia, Universidad Complutense de Madrid, 28040 Madrid, Spain; lmbedoya@ucm.es (L.-M.B.); naber@ucm.es (P.B.)

**Keywords:** HIV vaginal preexposure prophylaxis, dapivirine controlled release, bioadhesive vaginal tablets, pectin, locust bean gum, chitosan

## Abstract

Young sub-Saharan women are a group that is vulnerable to the sexual transmission of HIV. Pre-exposure prophylaxis through vaginal microbicides could provide them an option for self-protection. Dapivirine has been demonstrated to have topical inhibitory effects in HIV, and to provide protection against the sexual transmission of this virus. This paper reports on the studies into swelling behaviour, bioadhesion and release carried out on dapivirine tablets based on chitosan, locust bean gum and pectin, to select the most suitable formulation. The modified simulated vaginal fluid led to a high solubility of dapivirine and allowed the dapivirine release profiles to be characterized in sink conditions; this aqueous medium is an alternative to organic solvents, which are not a realistic option when evaluating systems whose behaviour varies in aqueous and organic media. Of the formulations evaluated, dapivirine/pectin tablets containing 290 mg of polymer and 30 mg of dapivirine present the most moderate swelling, making them the most comfortable dosage forms. Their high bioadhesive capacity would also allow the formulation to remain in the action zone and release the drug in a sustained manner, pointing to this formulation as the most promising candidate for future evaluations of vaginal microbicides for the prevention of HIV.

## 1. Introduction

According to the Joint United Nations Programme on HIV and AIDS (UNAIDS), 36.7 million people today are living with HIV, and 1.8 million are infected each year [1]; nearly half those infected are women. In sub-Saharan Africa, almost six out of 10 infected adults are women, and infected women 15–24 years old represent 76% of all the cases registered in that age range, with more than three infected women for each infected man [2,3]. Multiple concurrent relationships, limited skills in negotiating safer sex practices—condom use—and gender-based violence make women more vulnerable than men to infection through sexual intercourse [4]. New technologies are therefore needed that can be self-initiated by women to prevent the sexual transmission of HIV [2,3,4], but other factors need to be taken into account such as women’s preferences and the range of socioeconomic settings. Topical Preexposure Prophylaxis (PrEP) has been widely explored, including formulations containing anti-HIV drugs that are placed in the vagina with the aim of preventing sexual transmission through the mucosa in sexual encounters. These systems are generally known as vaginal microbicides [5,6], and allow the drug to reach the site of activity and remain in the vagina for an adequate period of time [7]. A product for this purpose must be cheap to manufacture, easy to administer, and comfortable once inserted [5,8]. Moreover, the microbicides must exhibit no mucosal toxicity, as inappropriate immune responses can actually facilitate HIV transmission [7,9]. To overcome these drawbacks, efforts have been made over the last 20 years to find an ideal microbicide that does not induce disruption in the vaginal epithelium, significantly modify the microbiota or induce inflammation [6,9].

Dapivirine (DPV) is a diarylpyrimidinic non-nucleoside reverse transcriptase inhibitor (NNTI) that inhibits viral replication. Topical DPV demonstrated potent dose-dependent inhibitory effects, with half-maximal inhibitory concentration [IC_50_] values in the nanomolar range (less than 5 ng/mL). Prolonged protection over several days after tissue incubation with DPV and a lower resistance induction compared to other NNTIs have been documented [5]. In an animal model, a 0.009% DPV gel showed concentrations in cervical and vaginal tissue over the 90% effective in vitro concentration up to 48 h later [7]. Some formulations have even reached clinical trials, and it has been shown that DPV administered in a vaginal ring reduced the risk of acquiring HIV by 31%, with no severe adverse effects detected [10,11].

Unfortunately, there are very few published results on the development of vaginal DPV systems, although different dosage forms such as rings [10,11], gels [12], diaphragms [13], films [14] and tablets [15] are currently being explored. Vaginal tablets offer many interesting properties, including their high stability, small size and low production costs compared to other controlled release systems [5]. They could therefore be useful for the controlled release of DPV using gelling and mucoadhesive polymers [8] like locust bean gum (LBG), pectin (P) and chitosan (CH) [16,17].

CH is a linear copolymer obtained by partial deacetylation of chitin (N-acetylglucosamine), which is obtained from the exoskeleton of crustaceans. CH is not soluble in water but forms a gel by adding diluted acids [18,19], and the percentage of deacetylation determines its physicochemical properties. These properties have led to its use in vaginal formulations such as films [20], tablets [21], liposomes [22], nanoparticles [23] and dendrimers [24], either alone or in combination with other polymers [25,26]. LBG is a galactomannan polymer extracted from the endosperm of the seeds of *Ceratonia siliqua L* [27], and is emerging as an excipient for the development of different controlled drug-release dosage forms due to its gelling, thickening and bioadhesive properties; it forms strong elastic gels, either alone or in combination with others [28,29]. Lastly, P is a heteropolysaccharide obtained from apple or citrus peel containing at least 65% galacturonic acid units, and is a promising tool for the development of various mucoadhesive formulations such as gels, tablets and moisturisers [16,30,31,32].

With this background, the aim of the present research was to develop natural polymer-based tablets with good bioadhesion propertied for the controlled release of DPV as a potentially promising option for preventing the sexual transmission of HIV in women.

## 2. Materials and Methods

### 2.1. Materials

Dapivirine (DPV, Lot:60416PIL04, MW = 329.4 Da) was a kind gift from the International Partnership for Microbicides (IPM, Silver Spring, MD, USA). According to previously published results, a low molecular weight chitosan (CH) was used (32 kDa), with a degree of deacetylation ≈55% (Lot:0055790, purchased from Guinama (Valencia, Spain)). The molecular weight of pectin (P) is 219 KDa and it is highly methoxylated, ≈80% (Lot:BCBK7271V, supplied by Sigma Aldrich (St. Louis, MO, USA)). As for locust bean gum (LBG), a molecular weight of 2780 KDa was found (Lot:010M0087, supplied by Sigma Aldrich) [17]. Sodium lauryl sulphate (SLS, Lot:STBG2381V) was purchased from Sigma Aldrich. All other reagents used in this study were of analytical grade and used without further purification. Demineralized water was used in all cases.

### 2.2. Methods

#### 2.2.1. Preliminary Studies 

##### Characterization of the Texture of Polymer Gels

As the polymers used to prepare the tablets jellify in water, which determines the release process [16], texture analyses were carried out to obtain the rheological characterization of P, LBG and CH gels in simulated vaginal fluid (SVF, pH = 4.2 [33]). 

Gels were prepared with increasing amounts of the polymers in SVF. To ensure the homogeneous distribution of the polymer in the medium, samples were stored at room temperature for 24 h, and the gels obtained were subsequently assessed using a TA.XT*plus* Texture Analyser (Stable Micro Systems, Godalming, UK). A 20 mm diameter stainless-steel probe with an activation force of 2 g was introduced in each gel at a rate of 0.5 mm/s to a depth of 15 mm, and returned to the initial height at the same rate. 500 points per second were monitored during data collection, which allowed the calculation of the penetration force, an accurate prediction of gel consistency. The detachment force, said to be a predictor of adhesive performance, was also recorded. The peaks for both indicators were then compared with the concentration of the polymer gels, thus obtaining their consistency and adhesiveness according to this variable. 

Maximum penetration force (MPF) and Maximum detachment force (MDF) for each polymer were then statistically processed through a two-way ANOVA considering polymer nature and concentration as factors (α = 0.05).

##### Dapivirine Solubility Tests

DPV is poorly soluble in water (<1 µg/mL), making it difficult to assess its release from the formulations [5]. There have been numerous attempts to adequately evaluate the release of DPV from different types of formulations, and most to date have used media based on isopropanol [13,34]. In this scenario, it was necessary to select a dissolution medium to overcome the low solubility of this drug in water in order to perform the release test in sink conditions. DPV solubility was therefore assessed in various aqueous media: namely, isopropanol or propanol/water mixtures, isopropanol and propanol/SVF mixtures and SLS in water and SVF dissolutions. An excess of DPV was added to 5 mL of various media in test tubes, which were then introduced in a shaking water bath (Selecta^®^ UNITRONIC320 OR, Barcelona, Spain) at room temperature and 15 rpm. The saturation of all the media was obtained after 72 h. The samples were filtered and the concentration of DPV was evaluated in a UV-visible spectrophotometer (Thermo Fisher Scientific^®^ Evolution 60S UV-Visible Spectrophotometer, Waltham, MA, USA). All the results were compared to the desired solubility by a unilateral *t*-student comparison (*H*_0_ → Solubility higher than desired, *H*_1_ → Solubility equal to or lower than desired), α = 0.05.

#### 2.2.2. Preparation of the Tablets 

Physical mixtures of the components shown in Table 1 were compacted at a pressure of 3.68·10^8^ Pa for 240 s, using a press similar to that used for the preparation of samples for the IR technique [35], with 13 mm diameter dies. All the samples obtained were stored in a desiccator until further analysis.

#### 2.2.3. Assessment of the Tablets 

##### Swelling Test

The swelling behaviour of the tablets in aqueous media is a critical factor when the main excipient is a swellable polymer, as it may be directly related with the process and rate of drug release and the mucoadhesive capacity of the systems. Swelling profiles over time were evaluated in all the batches. Three samples of each formulation were fixed to a stainless-steel disc and placed in beakers containing 150 mL of SVF, which were then introduced in a shaking water bath at 37 °C and 15 rpm [26].

The samples were weighed every hour—after removing the excess liquid—during the first six hours. The process was then repeated daily until the complete dissolution or erosion of the system. Each analysis was carried out in triplicate, and the swelling ratio (SR) was determined according to the Equation (1) [36]:

SR = [(*W_t_* − *W*_0_)/*W*_0_]·100, (1)

where *W_t_* and *W*_0_ correspond to the weight of the swollen tablet and the weight of the dry tablet respectively. Maximum swelling ratios (SR_max_) and areas under the curve (AUC) for each batch were then statistically processed through two-way ANOVA, considering polymer nature and drug content as factors (α = 0.05).

##### Drug Release Study

Each formulation was placed in the bottom of a borosilicate glass flask containing 80 mL of the selected medium to obtain sink conditions, then placed in a shaking water bath at 37 °C and 15 rpm. At given times, 5 mL were taken and immediately replaced with 5 mL of tempered medium. The aliquot was filtered and the concentration of DPV was determined by UV spectroscopy at a wavelength of 287.5 nm [21,26]. The study was performed in triplicate and the results were statistically processed to compare the batches using the similarity factor *f*_2_ [37].

The data from the release studies were adjusted to different kinetic models to determine the release mechanism of the drug; namely, zero-order (Equation (2)), Hopfenberg (Equation (3)), Higuchi (Equation (4)) and Korsmeyer‒Peppas (Equation (5)).

*M_t_* = *M*_0_ + *k*_0_*t*, (2)

*M_t_*/*M*_ꝏ_ = 1 − [1 − *k_HF_t*]*^nH^*, (3)

*M_t_*/*M*_ꝏ_ = *K_H_t*^1^^⁄^^2^, (4)

*M_t_*/*M*_ꝏ_ = *K_k_t^nK^*, (5)

where *M_t_* is the amount of drug released at a time *t*, *M*_0_ is the initial amount of drug released (in most cases, *M*_0_ = 0), *M*_∞_ is the total amount of drug in the compact and *M*_∞_/*M_t_* is the proportion of drug remaining in the compact at a time *t*. *K*_0_, *K_HF_ K_H_* and *K_KP_* are the release constant of the process for zero order, Hopfenberg, Higuchi and Korsmeyer‒Peppas kinetics respectively. *K_HF_* represents the equation *K*_0_/*C*_0_*a*_0_, in which *K*_0_ is the rate of erosion constant, *C*_0_ is the starting concentration of drug in the matrix and *a*_0_ represents the radius of the matrix. *nH* is the Hopfenberg release exponent, and takes values of 1, 2 and 3 for a brick, cylinder and sphere respectively. *nk* is the Korsmeyer‒Peppas release exponent, which characterizes the drug-release mechanism. The release may be produced either by pure diffusion (Fickian process) when *nk* ≤ 0.45, anomalous transport (combination of simultaneous processes of diffusion and relaxation of the polymer fibres) when 0.45 < *nk* < 0.89, or case II and super case II transport (where the release is governed by the relaxation of the polymer chains) when *nk* ≥ 0.89.

##### Bioadhesion Test

In vaginal controlled release, the dosage form must remain attached to the mucosa for the period required to release the drug. The bioadhesion force was therefore assessed using the TA.XT*plus* Texture Analyser (Stable Micro Systems, Godalming, UK). The samples were fixed to a 10-mm stainless-steel probe. A fragment of tanned goat skin was fixed to a methacrylate support rig [38] and immersed in a beaker with 500 mL of SVF. The probe with the sample was moved at a speed of 1 mm/s until it came into contact with the tanned skin, applying a contact force of 500 g for 30 s. The probe was then separated from the sample at a speed of 1 mm/s up to the starting height of the test. The force and work required to detach the formulation were measured at a rate of 500 pps. Each batch was evaluated in duplicate, and the results were statistically processed using two-way ANOVA, with drug content and polymer amount as factors (α = 0.05).

##### Cytotoxicity Evaluation

Two human cell lines were used: MT-2 [39] and HEC-1A. MT-2 cells are lymphoblastoid in origin and are derived from T-cells. HEC1A is a uterus/endometrium epithelial cell line (kindly provided by María Angeles Muñoz, Hospital Gregorio Marañón, Madrid, Spain). Cells were grown in RPMI 1640 medium supplemented with 10% (*v*/*v*) foetal bovine serum, 2 mM l-glutamine and 50 μg/mL streptomycin at 37 °C with a humidified atmosphere of 5% CO_2_. HEC-1A cells were detached by treatment with a trypsin 0.25% and EDTA 0.03% solution. Cell cultures were split twice a week.

Cell toxicity was measured by the MTT (3-[4,5-dimethylthylthiazol-2-yl]-2,5-diphenyltetrazolium bromide) method. Cells were incubated in 96-well plates at a density of 10 × 10^4^ cells per well in the case of MT-2; and 2 × 10^4^ in the case of HEC-1-A in complete RPMI medium. To assess the cytotoxic effects of the suspensions, cells were exposed to fresh medium containing various concentrations of the suspensions or PBS 1× (used to dissolve compounds) in triplicate for 48 h. A standard method was followed to suspend compounds in PBS 1× [26]. After an incubation of 48 h, 20 µL of MTT (Sigma-Aldrich) solution (7.5 mg/mL) was added to the plate and incubated for another two hours for MT-2 cells and three hours for HEC-1A cells. The supernatant was then carefully removed and 100 µL of dimethyl sulfoxide (DMSO, Sigma-Aldrich) was added to each well. Absorbance at a wavelength of 550 nm was measured in a microplate spectrophotometer (Labtech LT-4000, Heathfield, UK). Cytotoxic concentration 50 (CC_50_) values were calculated using GraphPad (San Diego, CA, USA) Prism Software (non-linear regression, log inhibitor versus response). The results of the MTT assay represented the average of at least three individual experiments.

## 3. Results and Discussion

### 3.1. Preliminary Studies 

#### 3.1.1. Characterization of the Texture of Polymer Gels

The maximum force needed to penetrate each gel was measured (Maximum Penetration Force, MPF). The probe was then extracted and the maximum force required to completely detach the gel was also recorded (Maximum Detachment Force, MDF). 

**Figure 1:**
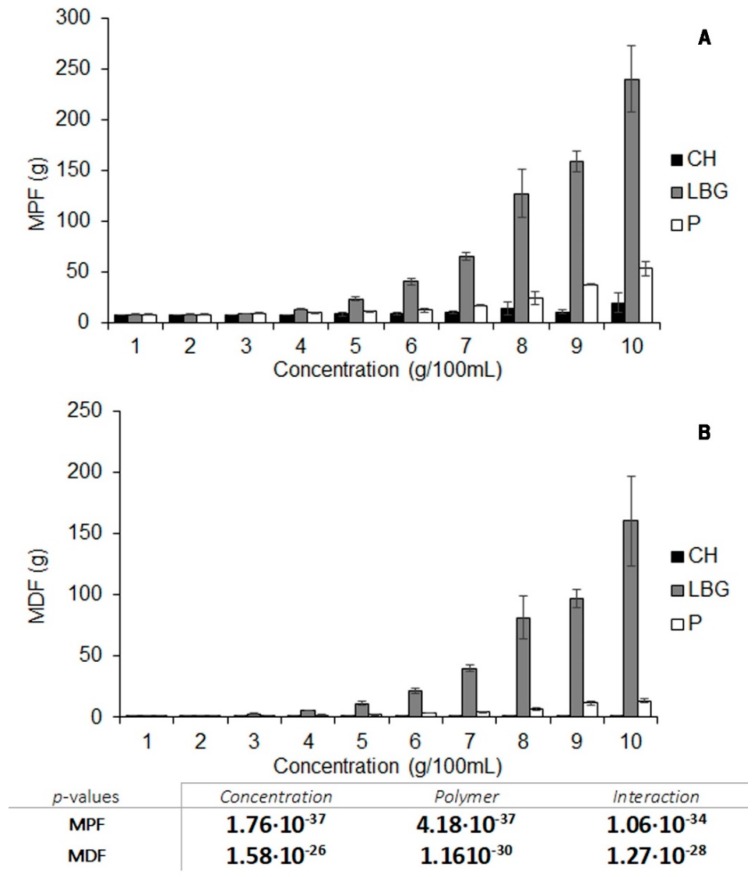
Maximum penetration force (**A**) and maximum detachment force (**B**) profiles for different concentrations of chitosan, pectin and locust bean gum gels in simulated vaginal fluid (SVF) evaluated in triplicate. *p*-Values obtained through two-way ANOVA processing are included below the graphics with polymer concentration and polymer nature as factors (α = 0.05). The bold font indicates significant differences.

MPF and MDF values for the three polymers in SVF are shown in Figure 1A,B, respectively. All the polymer gels revealed increasing values of MPF when the concentration of polymer was higher than 4 g/100 mL SVF, thus indicating that polymers jellify at this concentration or higher. MDF values also increased for LBG and P when the polymer concentration was over 4 g/100 mL, but remained constant for CH systems. CH was unable to generate a homogeneous gel in SVF as this medium does not meet the requirements; it has insufficient acidity to imbibe the polymer chains [18]. Nevertheless, it is sufficiently acid to break the bonds between the CH chains, and the imperfect gelation of the polymer produces a flocculation process in the medium, leading to a heterogeneous system with a higher flocculate height the greater the concentration of the polymer. When the amount of polymer was above 5 g/100 mL, a higher MDP was required to penetrate the flocculate, however, the three-dimensional system was unable to attach to the probe, hence the constant MDF values for all the CH systems. LBG can be said to generate the most consistent gel, and has the highest penetration and detachment rates. Gels are also obtained for P, but show lower consistencies, as can be seen from the texture analysis curves. These results indicate that LBG forms a much stronger gel in the medium than P. 

#### 3.1.2. DPV Solubility Tests

A DPV solubility of over 1.875 mg/mL was required to obtain sink conditions in the drug release study for the prepared dosage forms (30 mg DPV per tablet in 80 mL of dissolution medium), so solubility tests were done on DPV in different media.

**Figure 2:**
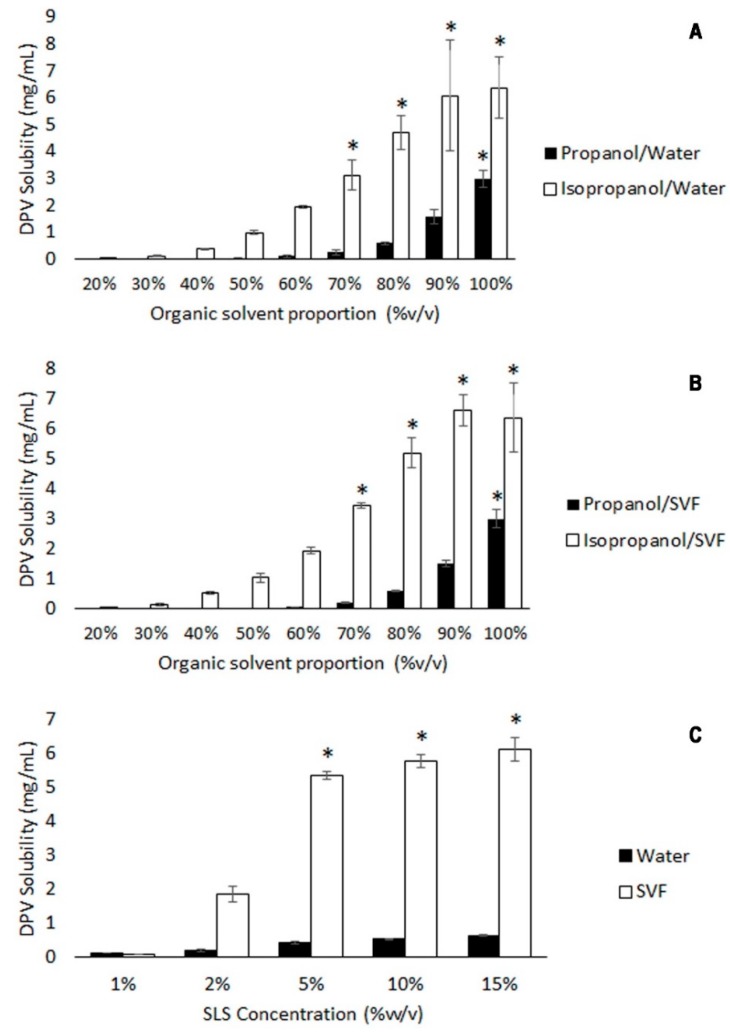
Dapivirine (DPV) solubility in isopropanol/propanol and water mixtures (**A**), isopropanol/propanol and simulated vaginal fluid (SVF) mixtures (**B**) and sodium lauryl sulphate/simulated vaginal fluid (SLS/SVF) and sodium lauryl sulphate/water (SLS/water) solutions (**C**) performed in triplicate. An asterisk on the bar indicates that the result is significantly greater than the solubility required for the study (α = 0.05).

The solubility data for DPV in pure isopropanol and propanol are 6.4 mg/mL and 3.0 mg/mL, respectively. These results agree with the previously published data [34]. Although isopropanol could be a good medium for DPV release studies in sink conditions due to its high solubility in this solvent, we have shown that the tablets in this research failed to jellify in either solvent. DPV solubility was therefore assessed in binary mixtures of isopropanol or propanol with water or SVF, and in micellar solutions of SLS in water and SVF. Figure 2A,B show that the addition of increasing amounts of any of the aqueous media to isopropanol or propanol drastically decreases DPV solubility, indicating the clear influence of dielectric constant values (ε) on DPV solubility –ε(isopropanol) = 18.62, ε(propanol) = 22.20 and ε(water) = 78.54– [40]. The results in Figure 2C point to the solubilising power of SLS in water or SVF, and DPV solubility increases with higher concentrations of SLS. SLS has higher solubilising power in SVF, and these results may be in agreement with das Neves et al., who report that DPV solubility increases as pH decreases [5]. This also conditions the release profiles, since it has been verified that 5% SLS in water has a pH value of 7.2, while 5% SLS in SVF still has a pH value of 4.2, emulating the acid conditions of the vaginal environment. It was also observed that these micellar solutions allow the DPV tablets to jellify, while propanol and isopropanol aqueous mixtures do not. 

In view of these results, SVF containing 5% SLS—where DPV solubility is above 5 mg/mL—was selected as the medium (henceforth modified SVF (mSVF)) to evaluate DPV release, as it represents a suitable option for the evaluation of the natural polymer-based vaginal tablets, due to the fact that sink conditions (DPV maximum concentration = 1.87 mg/mL) are widely exceeded and polymers are able to jellify.

### 3.2. Assessment of the Tablets 

#### 3.2.1. Swelling Test

**Figure 3:**
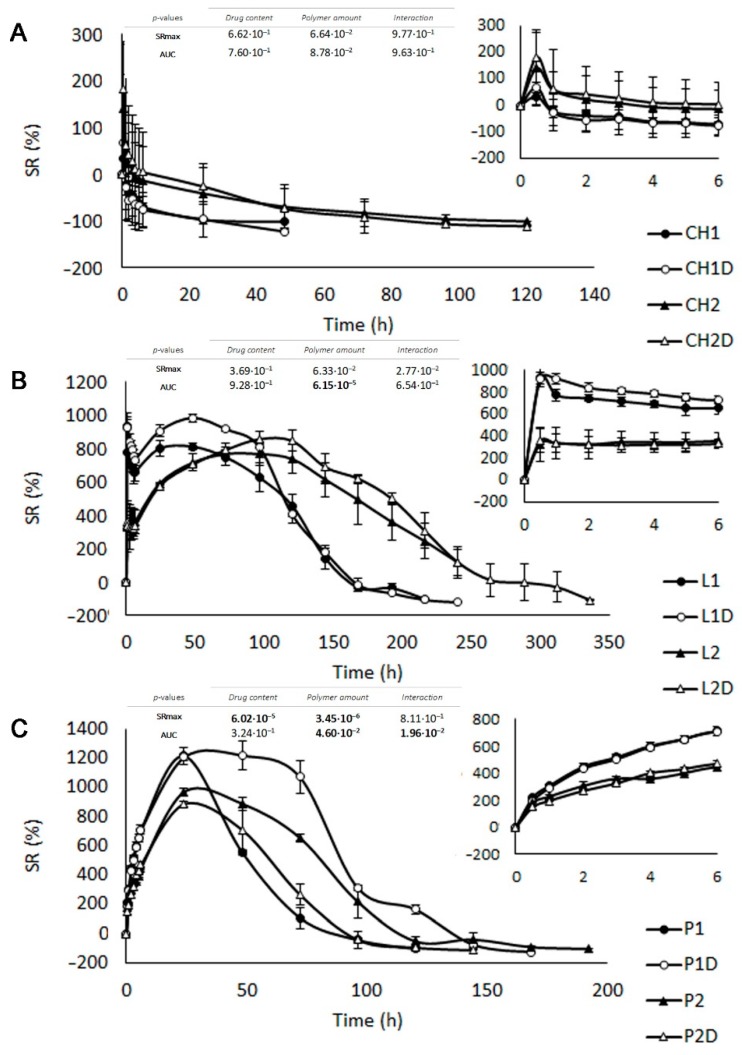
Swelling behaviour profiles in simulated vaginal fluid (SVF) for chitosan (**A**), locust bean gum (**B**) and pectin (**C**) batches evaluated in triplicate. The first six hours are shown in detail in the graphics in the bottom right corner. *p*-Values obtained for SR_max_ and AUC through two-way ANOVA processing are included in the graphics with polymer amount and drug content as factors (α = 0.05). The bold font indicates significant differences.

Figure 3A shows the swelling test profiles from CH batches. All samples underwent moderate swelling in the first 30 min of the assay, followed by an erosion process. This behaviour is due to the inability of CH to form a gel in SVF, as has been previously described in the texture analysis results. Slight differences can be seen in the swelling curves of the batches containing different amounts of CH. Batches containing a higher amount of polymer (CH2 and CH2D) take longer to erode totally (120 h) due to the difference in the tablet’s surface/polymer amount ratio (s/a). It can also be seen that, although DPV is highly hydrophobic, the presence of the drug does not modify the erosion process. 

Figure 3B shows the swelling curves of LB systems. The amount of polymer in each sample clearly conditions the *t_max_*; when the amount of polymer is increased, *t_max_* is also prolonged and occurs in 48 h for L1 and 96 h for L2 (Table 2). Although the maximum swelling is similar in all cases, tablets containing a higher amount of polymer take longer to totally erode. These curves can be justified by the fact that swelling and erosion occur simultaneously, as in P- and CH-based tablets. In terms of *t_max_* values, erosion of a complete swelled gel occurs in L1 while a more pronounced plateau is observed in the swelling curve for L2; the erosion also takes longer to complete (312 h) and occurs concurrently with swelling. The addition of DPV to the tablets does not modify LBG swelling behaviour, suggesting that the arrangement of the polymer chains in the aqueous medium is not altered by the presence of hydrophobic particles in the drug. Finally, LB tablets disaggregate in small gel particles in the medium and gradually lose their structure.

Figure 3C shows the swelling curves for P tablets. A swelling and erosion process were detected in every case. The time taken to achieve maximum swelling (*t_max_*) is in the same range (24–48 h), although differences can be seen in the total proportion of water imbibed and in the time required for complete erosion, quantified by the area under the curve (*AUC*). The amount of P in the batches conditions the swelling behaviour, which is more pronounced the less polymer there is in the tablet (P1), although erosion is also faster. This can be attributed to the *S*/a ratio, as the larger the surface of the tablet in contact with water, the more water penetration is favoured, producing a more fluid gel than for P2 and P2D. The content of DPV in P1D and P2D confers some hydrophobia on the tablets and also slightly increases the S/a ratio, reducing the surface in contact with the medium. Consequently, P1D and P2D batches imbibed a smaller amount of SVF than P1 and P2.

#### 3.2.2. Drug Release Study

**Figure 4:**
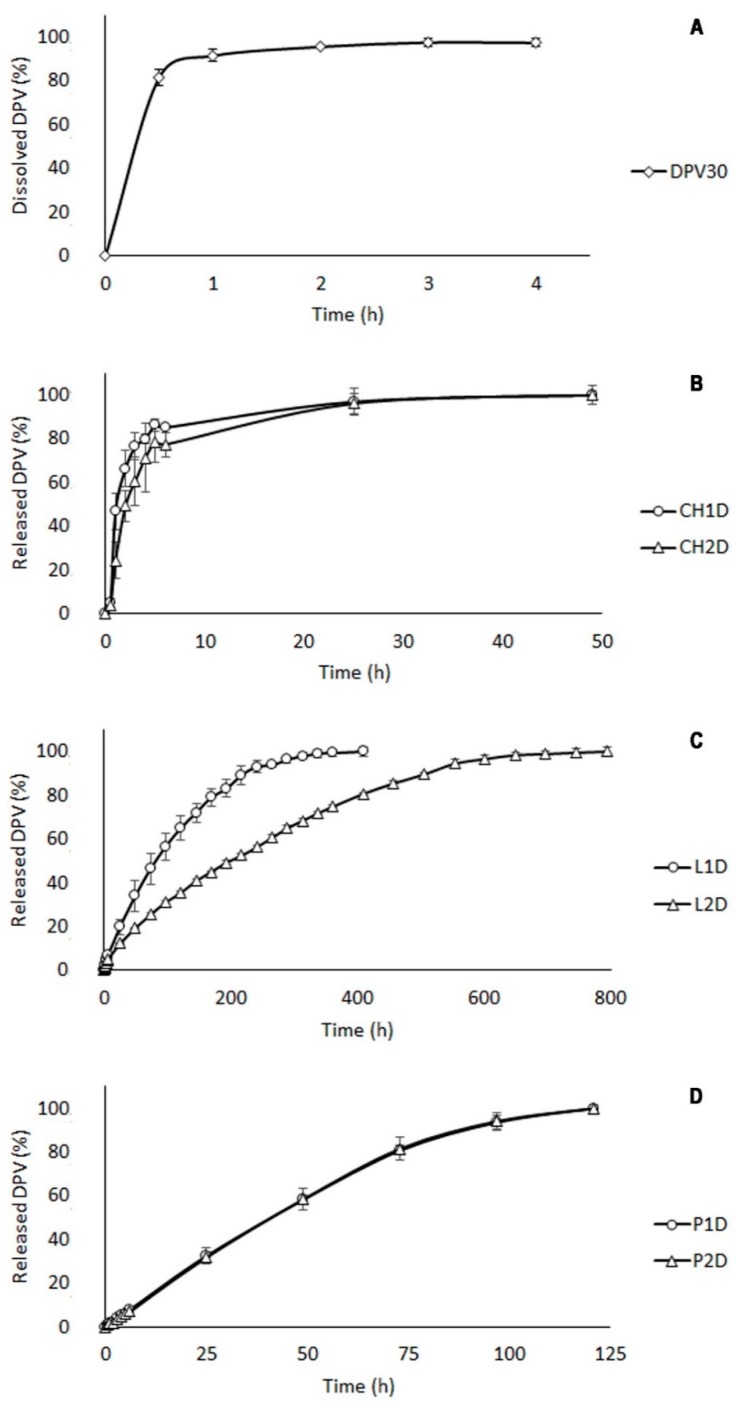
Dapivirine (DPV) dissolution profile (**A**) and Dapivirine (DPV) release profiles from chitosan (**B**), locust bean gum (**C**) and pectin (**D**) batches in modified simulated vaginal fluid (mSVF) evaluated in triplicate.

The dissolution process of pure micronized DPV was assessed in mSVF (Figure 4A) and revealed the almost complete dissolution of the drug (93%) over a period of 4 h. DPV release profiles from polymer systems are different, and a controlled release was obtained in all cases, although the release profile clearly varies depending on the polymer used. Differences were observed in the drug release between CH batches (CH1D and CH2D) during the first six hours, but the rapid erosion of the tablets led to a quick and complete DPV release in the first 24 h for both batches (Figure 4B). 

LBG is the polymer that most closely controls the release of DPV from the batches (L1D and LD2). In the case of batch L1D, up to 410 h are required to release the entire dose. In the case of batch L2D, the drug release extended to 792 h, or 33 days (Figure 4C). 

Batches based on P (P1D and P2D) released the entire drug amount in 120 h in a sustained manner with overlapping profiles (Figure 4D), indicating that P systems are sufficiently robust to release DPV in a sustained and uniform manner in the proportions evaluated in this study. 

However, vaginal turnover must be taken into account. This is a physiological mechanism whose objective is to remove possible foreign elements from the environment of the vaginal epithelium (such as pathogens or any extraneous element). This represents a limitation for these systems, as this cyclical process takes place in 96 h [41]. Vaginal leakage limits the efficiency of L tablets during such prolonged periods in in vivo studies; they would be expelled when no more than 60% of the dose had been released. In contrast, P systems release over 90% of the drug in this period, so vaginal clearance would occur after the release of almost the entire dose of the drug. It should also be noted that this is an in vitro testing of the systems, so vaginal discharge must be considered in future research stages, as this causes the loss of the drug, leading the concentration in vaginal tissue to be lower than predicted.

It should be noted that swelling results are related to the drug release profiles: the higher the AUC and *t_max_* for a formulation, the more controlled is the release of the drug. 

The results obtained when comparing the batches using an *f*_2_ statistical process (Table 3) highlight significant differences in the release of DPV in all the systems, except between the two batches constituted by P, whose release profiles can be considered to be overlapping. 

Mathematical models were used to determine the drug release mechanisms [42]. Table 4 shows the results for these fits.

While CH batches do not fit any kinetic model, P and L batches can be adjusted to Korsmeyer‒Peppas with a good correlation coefficient. In the case of L batches, a good adjustment to this model with a *nk* value of between 0.45 and 0.89 implies the release of the drug through the simultaneous relaxation of the polymer chains and Fickian diffusion processes, agreeing with the data obtained from the swelling studies, as this polymer simultaneously undergoes swelling and erosion during the release process. This can be confirmed by the fact that these systems also show a good fit to Hopfenberg and Higuchi kinetics, indicating erosion and diffusion as their respective release mechanisms. P batches have higher values of *nK* for adjustment to the Korsmeyer‒Peppas kinetic, suggesting that the release occurs mostly through polymer chain relaxation. This is confirmed by the higher correlation coefficient observed for Hopfenberg (erosion) compared to Higuchi (diffusion). These results also agree with the swelling studies, as P erodes rapidly after reaching its maximum while the drug is still being released.

It is also worth highlighting that systems P1D and P2D release DPV in the medium at a constant rate during the first 72 h, so the dissolution of the drug from the system occurs at the same rate the drug is released. This is explained by the fact that the release occurs through an erosion process in which the pectin acts throughout this period as a purely erodible matrix (Costa & Lobo, 2001), leading to a pseudo-zero-order process where the release of the drug would lead to constant concentrations of DPV in the vagina while the formulation remains attached to the mucosa, thus avoiding toxic or ineffective concentrations.

**Figure 5:**
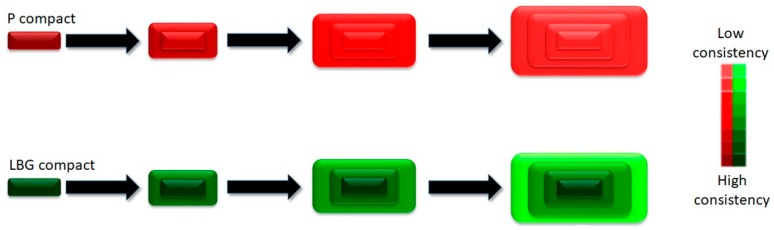
Evolution of pectin- and locust bean gum-based tablets in contact with simulated vaginal fluid (SVF). The pectin tablet (red) exhibits uniform swelling and produces a gel with a homogeneous consistency. The locust bean gum tablet (green) swells abruptly and generates a gel with a heterogeneous consistency.

Drug release data are related to texture behaviour: the consistency acquired by P tablets in contact with SVF is not dependent on the amount of P. The opposite occurs in the case of LBG, so an increase in the amount of polymer reduces the amount of drug released. This can be explained by the fact that gel texture analyses (Section 3.1.1) revealed that an increase in P concentration slightly increased the consistency parameters (MPF and MDF), in contrast with LBG-based gels. The consistency of the swelled P tablets is therefore homogeneous, while in LBG-based tablets it is heterogeneous and greater at the deepest layers (Figure 5), hence the differences between the release profiles for the L batches and the homogeneity between P batches.

Although vaginal rings that show a longer release of DPV have been reported previously [43], the technologies and excipients required to their manufacturing make them too expensive. Taking into account that these formulations have been designed for use in developing countries, the reduction in manufacturing costs may imply greater access to pre-exposure prophylaxis therapies. According to this, studies on the effectiveness of our tablets can be justified in future research once the drug has proved capable of release from the formulation in the swollen tablets.

#### 3.2.3. Bioadhesion Test

**Figure 6:**
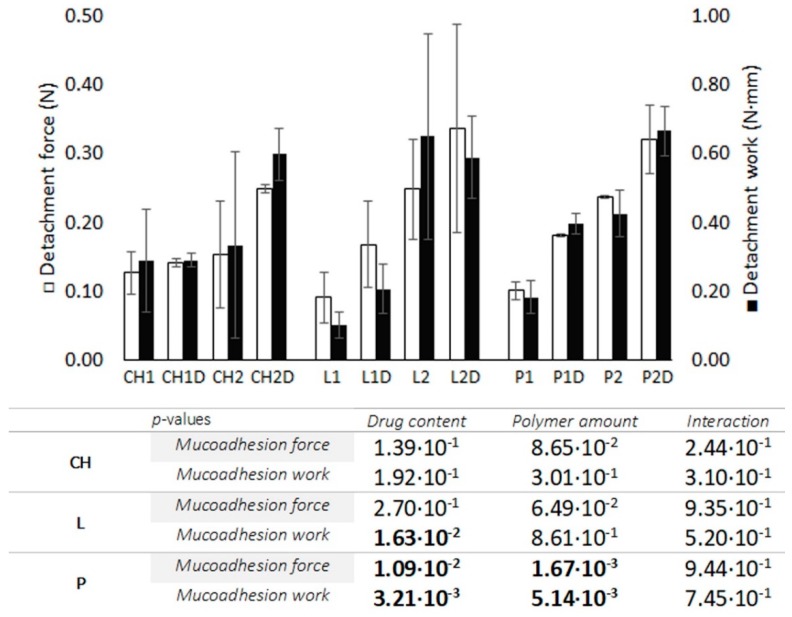
Bioadhesion force and work in chitosan-, locust bean gum- and pectin-based blank and loaded batches, evaluated in duplicate in tanned goat skin. *p*-Values obtained for mucoadhesion force and work through two-way ANOVA processing are included below the graphic with polymer amount and drug content as factors (α = 0.05). The bold font indicates significant differences.

Figure 6 shows the results of the bioadhesion test. CH batches have low bioadhesion values (between 0.12 and 0.25 N), and a rise in the value of this parameter can be seen when the proportion of CH in the system is increased. It is interesting to note that the addition of the drug reduces the variability with respect to the reference tablets, as the hydrophobic drug generates more compact structures and displays more uniform erosion. In LBG systems, an increase in the amount of polymer produces a greater adhesion force in the system, and these values are greatest when the drug is incorporated. Nevertheless, both L2 batches show a wide variability between the samples due to the heterogeneity of the gel, as seen in Section 3.1.1. Similar results for detachment force and work are obtained when analysing systems containing P, where bioadhesion forces increase as greater amounts of polymer are added to the system. The addition of DPV also enhances bioadhesion forces in all the cases. This is probably due to the S/a ratio, as an increase in the amount of polymer leads to a higher proportion per unit of area, thus impeding the penetration of water and facilitating contact between the biological surface and the polymer. Likewise, the incorporation of DPV increases the systems hydrophobicity, resulting in less hydrated gels with a greater bioadhesion capacity; the highest bioadhesion force values are obtained in the system containing DPV and a higher proportion of polymer. These results suggest that the interactions between the polymer chains and the aqueous medium after gelation compete with the interactions taking place between the bioadhesive polymer and the biological surface [44]. The consistency of the bioadhesive bonds must also be considered, as this plays a key role. Diluted gels have a lower interaction than concentrated gels when in contact with the mucosa, as diluted gels tend to slip and become detached. The tablets containing higher amounts of polymer are therefore more bioadhesive as they generate more consistent gels. This explains why gels have poorer adhesion than solid systems.

Statistical processing indicates that there cannot be said to be any statistically significant differences between CH batches in terms of either the amount of polymer or the presence of the drug. The same is true of L batches, due to the high variability in the bioadhesion values for L or CH batches, indicating that these systems may have a somewhat erratic adhesion to the area of action. However, there are significant differences in both factors in the case of P batches, where the variability between the samples is low in all cases; this implies that the adhesion of the tablets could be highly reproducible, so P can be considered a promising candidate for the development of mucoadhesive dosage forms [45]. No significant interaction was observed between the presence of drug and the amount of polymer in any of the batches, indicating that the drug’s contribution to the bioadhesion capacity is not conditioned by the amount of polymer.

The results of bioadhesion are a good indicator of the ability of the tablets to remain attached to the mucosa. This justifies future evaluations to check the in vivo permanence of the formulations in the vaginal environment.

#### 3.2.4. Cytotoxicity

The biocompatibility of the tablets was evaluated through an in vitro cell toxicity assay. All the components of the different batches were incubated with PBS 1× in humidified atmosphere at 37 °C and 5% CO_2_ for five days before the assay to ensure that any potential toxic component would be present in the suspension. MT-2, a lymphoblastoid cell line, and HEC-1A, a uterus-derived cell line, were seeded and treated with different dilutions of the suspensions. All the components were tested at a maximum concentration of 1000 μg/mL in base-5 serial dilutions. Experiments were performed on MT-2 cells to evaluate toxicity on the immune cells present in vaginal or uterine mucosae and on the uterine epithelial cell line (HEC-1A) to assess any potential damage to mucosae integrity. CC_50_ were calculated when possible.

As shown in Table 5, no toxicity was detected at the concentrations tested for the polymers or at the higher concentration of 1000 µg/mL.

DPV showed high cytotoxicity, and significantly higher for MT-2 than for HEC-1A. However, after complete dissolution of the entire dose in the evaluation medium, and even without considering the vaginal clearage rate (as it is an in vivo parameter), it would generate a 1 mM solution, a concentration that shows good compatibility with ex vivo human cervical tissue explants, as shown in the study by Fletcher et al. [46]. This can be attributed to the fact that vaginal tissue may protect these cells from toxic concentrations of the drug and act as a barrier to penetration. Further in vivo studies should therefore be done to determine whether these formulations present significant toxicity or not.

These results would justify the in vivo evaluation of the tablets, since the polymers showed no toxicity and the dose of dapivirine in the vaginal environment would not be able to produce tissue damage.

## 4. Conclusions

Dapivirine tablets based on natural polymers have been shown to be promising tools for the prevention of sexual transmission of HIV due to their gelling properties, bioadhesion capacity, and lack of toxicity in the area of action. 

The modified SVF led to a high solubility of DPV and allowed the characterization of DPV release profiles in sink conditions; this aqueous medium is an alternative to organic solvents, which are not a realistic option when evaluating systems whose behaviour varies in aqueous and organic media. 

Of the three polymers assessed, P tablets showed a moderately prolonged swelling over time and allowed the sustained release of the drug for up to five days, with a highly reproducible bioadhesion strength. Moreover, DPV release profiles did not depend on the amount of polymer in the formulation, so the robustness of the tablets allows the choice of the most appropriate formulation to be chosen in terms of comfort and bioadhesion.

P can therefore be highlighted as an interesting option for the development of vaginal tablets for the controlled release of DPV. The most suitable candidates are the tablets containing 290 mg of polymer and 30 mg of DPV, as they present the most moderate swelling, making them the most comfortable dosage forms. Their high bioadhesive capacity would also allow the formulation to remain in the action zone and release the drug in a sustained manner; they are therefore the most promising subject for future evaluations in the field of vaginal microbicides for the prevention of HIV.

## Figures and Tables

**Table 1:** Tablets’ composition.

Batch	DPV (mg)	CH (mg)	P (mg)	LBG (mg)	MgSt (mg)
CH1		145			3
CH2		290			3
CH1D	30	145			3
CH2D	30	290			3
L1				145	3
L2				290	3
L1D	30			145	3
L2D	30			290	3
P1			145		3
P2			290		3
P1D	30		145		3
P2D	30		290		3

**Table 2:** Swelling data of chitosan, pectin and locust bean batches in SVF.

Batch	S_max_ (%)	*t_max_* (h)	*t_erosion_* (h)	AUC (%·h)	*S*/a (mm^2^/g)
CH1	32.91	0.5	48	7.295	1.17
CH2	138.57	0.5	120	40.224	1.03
CH1D	54.32	0.5	48	11.647	0.70
CH2D	162.51	0.5	120	46.494	0.68
L1	793.64	48	216	1103.871	1.20
L2	758.05	96	312	1587.312	1.06
L1D	799.63	48	240	1078.568	0.73
L2D	766.71	96	360	1626.576	0.69
P1	1191.71	24	120	696.036	1.20
P2	960.91	24	192	782.980	1.06
P1D	993.50	72	168	1001.369	0.73
P2D	796.31	24	144	594.549	0.67

**Table 3:** Results obtained through *f_2_* comparison of drug release from the batches.

Batch	CH1D	CH2D	L1D	L2D	P1D	P2D
CH1D		43.16	13.72	13.44	13.71	13.57
CH2D			14.84	14.30	14.84	14.68
L1D				36.48	43.83	43.58
L2D					33.52	33.37
P1D						97.61
P2D						

**Table 4:** Results obtained through kinetics adjustment.

		CH1D	CH2D	L1D	L2D	P1D	P2D
Zero Order	*K*_0_	0.0084	0.0101	0.0029	0.0018	0.0090	0.0087
	*R* ^2^	0.3361	0.4755	0.9011	0.9779	0.9723	0.9714
Hopfenberg	*K_HP_*	0.0103	0.0113	0.0027	0.0016	0.0080	0.0081
	*R* ^2^	0.6162	0.7402	0.9873	0.9844	0.9973	0.9978
Higuchi	*K_H_*	0.3863	0.3603	0.0590	0.0380	0.0847	0.0821
	*R* ^2^	0.6221	0.8866	0.9815	0.9908	0.9389	0.9322
Korsmeyer‒Peppas	*n_K_*	1.8712	1.4864	0.8126	0.6597	0.9249	0.9511
	*K_KP_*	0.2431	0.1478	0.0150	0.0151	0.0151	0.0134
	*R* ^2^	0.8480	0.9155	0.9895	0.9992	0.9957	0.9895

**Table 5:** CC_50_ values of DPV, CH, P and LBG obtained from the cytotoxicity assay in MT-2 and HEC-1A cell lines.

Drug	Cell Line	CC50
DPV	MT-2	1.9 µg/mL
	HEC-1A	11.2 µg/mL
CH	MT-2	>1000 µg/mL
	HEC-1A	>1000 µg/mL
LBG	MT-2	>1000 µg/mL
	HEC-1A	>1000 µg/mL
P	MT-2	>1000 µg/mL
	HEC-1A	>1000 µg/mL
